# Diphenyl ditelluride anticancer activity and DNA topoisomerase I poisoning in human colon cancer HCT116 cells

**DOI:** 10.18632/oncotarget.28465

**Published:** 2023-06-21

**Authors:** André Luiz Mendes Juchem, Cristiano Trindade, Juliana Bondan da Silva, Miriana da Silva Machado, Temenouga Nikolova Guecheva, Jaqueline Cesar Rocha, Jenifer Saffi, Iuri Marques de Oliveira, João Antonio Pêgas Henriques, Alexandre Escargueil

**Affiliations:** ^1^Department of Biophysics/Postgraduate Program in Genetics and Molecular Biology, Federal University of Rio Grande do Sul, Porto Alegre, Brazil; ^2^Sorbonne Université, INSERM, Centre de Recherche Saint-Antoine, Paris F-75012, France; ^3^Department of Basic Health Sciences, Federal University of Health Sciences of Porto Alegre – UFCSPA, Porto Alegre - RS, Brazil; ^4^Department of Postgraduate Program in Molecular and Cell Biology Applied to Health, Lutheran University of Brazil (ULBRA), Canoas, Brazil; ^5^Institute of Molecular Biology, Bulgarian Academy of Sciences, Sofia, Bulgaria; ^6^Postgraduate Program in Biotechnology and Medical Sciences, University of Vale do Taquari - UNIVATES, Lajeado - RS, Brazil; ^*^These authors contributed equally to this work

**Keywords:** colorectal cancer, HCT116, diphenyl ditelluride, organotellurium, topoisomerase I

## Abstract

Diphenyl ditelluride (DPDT) is an organotellurium (OT) compound with pharmacological properties, including antioxidant, antigenotoxic and antimutagenic activities when applied at low concentrations. However, DPDT as well as other OT compounds also show cytotoxicity against mammalian cells when treatments occur at higher drug concentrations. Considering that the underlying mechanisms of toxicity of DPDT against tumor cells have been poorly explored, the objective of our study was to investigate the effects of DPDT against both human cancer and non-tumorigenic cells. As a model, we used the colonic HCT116 cancer cells and the MRC5 fibroblasts. Our results showed that DPDT preferentially targets HCT116 cancer cells when compared to MRC5 cells with IC_50_ values of 2.4 and 10.1 μM, respectively. This effect was accompanied by the induction of apoptosis and a pronounced G2/M cell cycle arrest in HCT116 cells. Furthermore, DPDT induces DNA strand breaks at concentrations below 5 μM in HCT116 cells and promotes the occurrence of DNA double strand breaks mostly during S-phase as measured by γ-H2AX/EdU double staining. Finally, DPDT forms covalent complexes with DNA topoisomerase I, as observed by the TARDIS assay, with a more prominent effect observed in HCT116 than in MRC5 cells. Taken together, our results show that DPDT preferentially targets HCT116 colon cancer cells likely through DNA topoisomerase I poisoning. This makes DPDT an interesting molecule for further development as an anti-proliferative compound in the context of cancer.

## INTRODUCTION

Colorectal cancer (CRC) is the third most commonly diagnosed malignancy and the second leading cause of cancer death worldwide [1]. The CRC progresses through a series of clinical and histopathological stages ranging from benign tumors (polyp) to malignant cancers (carcinoma) [2]. The initial treatment is surgery and possibly followed by chemotherapy [3–5]. Some drugs commonly used for CRC include: oxaliplatin (Eloxatin), 5-fluorouracil (5-FU), capecitabine (Xeloda) and irinotecan (CPT-11 or Camptosar) [6]. The combination regimens FOLFIRI (leucovorin, 5-fluorouracil, and irinotecan) or FOLFOXIRI (leucovorin, 5-fluorouracil, oxaliplatin and irinotecan) are considered as the standard of care for first-line treatment of patients with metastatic colorectal cancer (mCRC) [7–10]. Irinotecan is a topoisomerase I (TOP1) inhibitor, analogue to camptothecin (CPT), which inhibits the DNA religation step in the ternary covalent TOP1–DNA–drug complex. Irinotecan is therefore classified as a TOP1 “poison” as opposed to TOP1 “suppressors” (or catalytic inhibitors), which are inhibiting the ability of TOP1 to cleave DNA [11, 12]. Irinotecan triggers cell death by trapping TOP1 onto DNA, thus generating cytotoxic protein-linked DNA breaks [10, 11]. TOP1 is an important target of antitumor drugs and its inhibitors are widely used in clinical practice [11]. However, to date, only four TOP1 inhibitors have been approved by FDA: topotecan, irinotecan, belotecan, and trastuzumab deruxtecan [13, 14]. Importantly, the use of these compounds is limited by its inherent toxicity and their adverse effects in patients, such as diarrhea and neutropenia, might be dose-limiting, leading to treatment interruption [11, 15]. Moreover, the emergence of drug resistance in tumor cells remains a major problem and new TOP1 inhibitors should therefore be investigated [10, 11, 16–18]. In this context, we previously demonstrated that diphenyl ditelluride (DPDT), an otherwise solid and non-volatile organotellurium (OT) compound, which is often used as an intermediate in organic synthesis reactions, interacts with TOP1 in *Saccharomyces cerevisiae* as well as in a cell free system [19, 20]. Moreover, DPDT was reported to induce cytotoxic effects in human colon carcinoma (HT-29), heterogeneous human epithelial colorectal adenocarcinoma cells (Caco-2), ileocecal adenocarcinoma (HCT-8), melanoma (MDA-MB-435), glioblastoma (SF-295, U87 and U251) and promyelocytic leukemia (HL-60) cells [21–23]. Additionally, previous studies showed cytotoxic, genotoxic and mutagenic properties of DPDT in Chinese hamster fibroblast (V79) cells, *Salmonella typhimurium* and strains of yeast *S. cerevisiae* proficient and deficient in several DNA repair pathways [20, 24]. Interestingly, at non-cytotoxic concentrations, DPDT is able to prevent oxidative stress and DNA damage induced by several agents in V79 cells [25]. OT compounds could therefore induce a variety of toxic and/or non-toxic effects depending on the dose employed. This class of molecules has been pointed out as promising and useful alternatives by the pharmaceutical industry. In particular, the OT compounds showed anti-inflammatory, immunomodulatory, hepato- and neuroprotective properties as well as anticancer properties [19]. In that sense, 2,2′-dimethoxydiphenyl ditelluride, 2,2′-diamino-3,3′,5,5′-tetramethyldiphenyl ditelluride and 4,4′-diisopropyldiphenyl ditelluride showed anti-proliferative activities against HL-60 cells. Organotellurates also act as potential antitumor agents, by either directly inhibiting tumor cell proliferation, migration and invasion, or disrupting angiogenesis evaluated in murine melanoma model [26]. Finally, the OT compound AS101 demonstrates anti-tumor properties *in vitro* and *in vivo*, possibly due to its immunomodulatory activity [27].

Considering that the underlying mechanisms of toxicity of DPDT in tumor cells have been poorly explored, the objective of the present study was to investigate the effects of DPDT in both CRC (HCT116) and non-tumor (MRC5) cells. For this purpose, we evaluated how DPDT might affect cell survival and the cell cycle progression and we determined the capability of DPDT to induce apoptosis as well as to form DNA double strand breaks (DSB) in replicative cells and to inhibit TOP1. Finally, the accumulation of reactive oxygen species (ROS) was measured in both HCT116 and MRC5 cells. Together, our results will help to better define DPDT as a potential drug candidate for treating CRC.

## RESULTS

### DPDT shows cytotoxic activity against HCT116 and MRC5 cells

**Figure 1:**
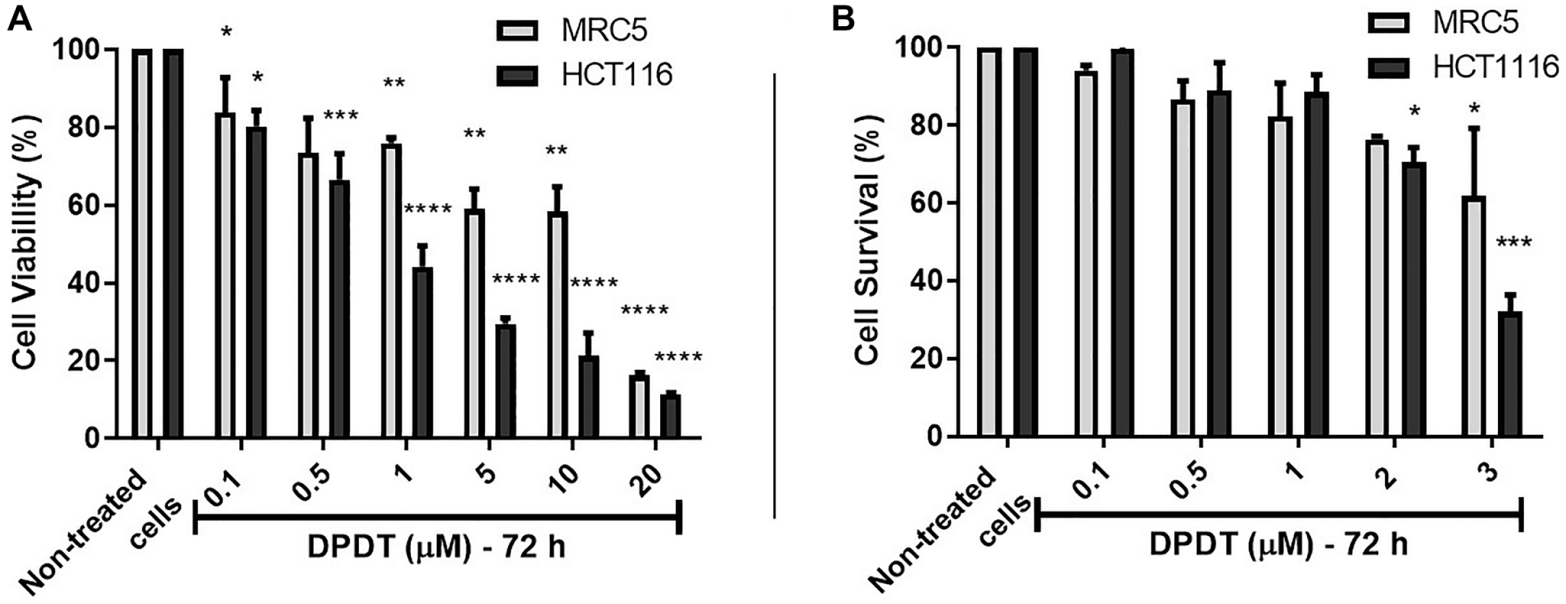
Cell viability assay of DPDT on Human MRC5 and HCT116 cell lines. (**A**) HCT116 and MRC5 cells were treated for 72 hours with increasing concentrations of DPDT (range: 0.1-20 μM) and cell viability assessed by the MTT assay. (**B**) HCT116 and MRC5 cells were treated for 72 hours with increasing concentrations of DPDT (range: 0.1–3 μM) and clone survival assessed by the colony-formation assay. All values are averages of at least 3 independent experiments done in duplicate. *P* values relative to the untreated control cells were calculated using one-way ANOVA Dunnett’s multiple comparison test: ^*^ *p* < 0.05, ^**^ *p* < 0.01, ^***^ *p* < 0.001, ^****^ *p* < 0.0001.

MRC5 and HCT116 cell lines were first treated for 72 hours with increasing concentrations of DPDT and the cell viability assessed through the MTT assay (Figure 1A). Our results demonstrated that DPDT preferentially targets HCT116 cells with an IC50 value of 2.4 μM against 10.1 μM for the non-tumorigenic MRC5 cell line. Because cell viability assays have limitations, the effect of DPDT was also evaluated through the colony formation assay (Figure 1B). Again DPDT more strongly inhibited the colony forming ability of HCT116 cells (IC_50_ = 2.80 μM) when compared to MRC5 cells (IC_50_ = 6.75 μM). Interestingly, the effect of DPDT was not limited to HCT116 cancer cell line since DPDT also acts on HeLa cells with a more pronounced effect than for HCT116 cells (Supplementary Figure 1).

### Exposure to DPDT induces caspases-mediated apoptosis in HCT116 cells

**Figure 2:**
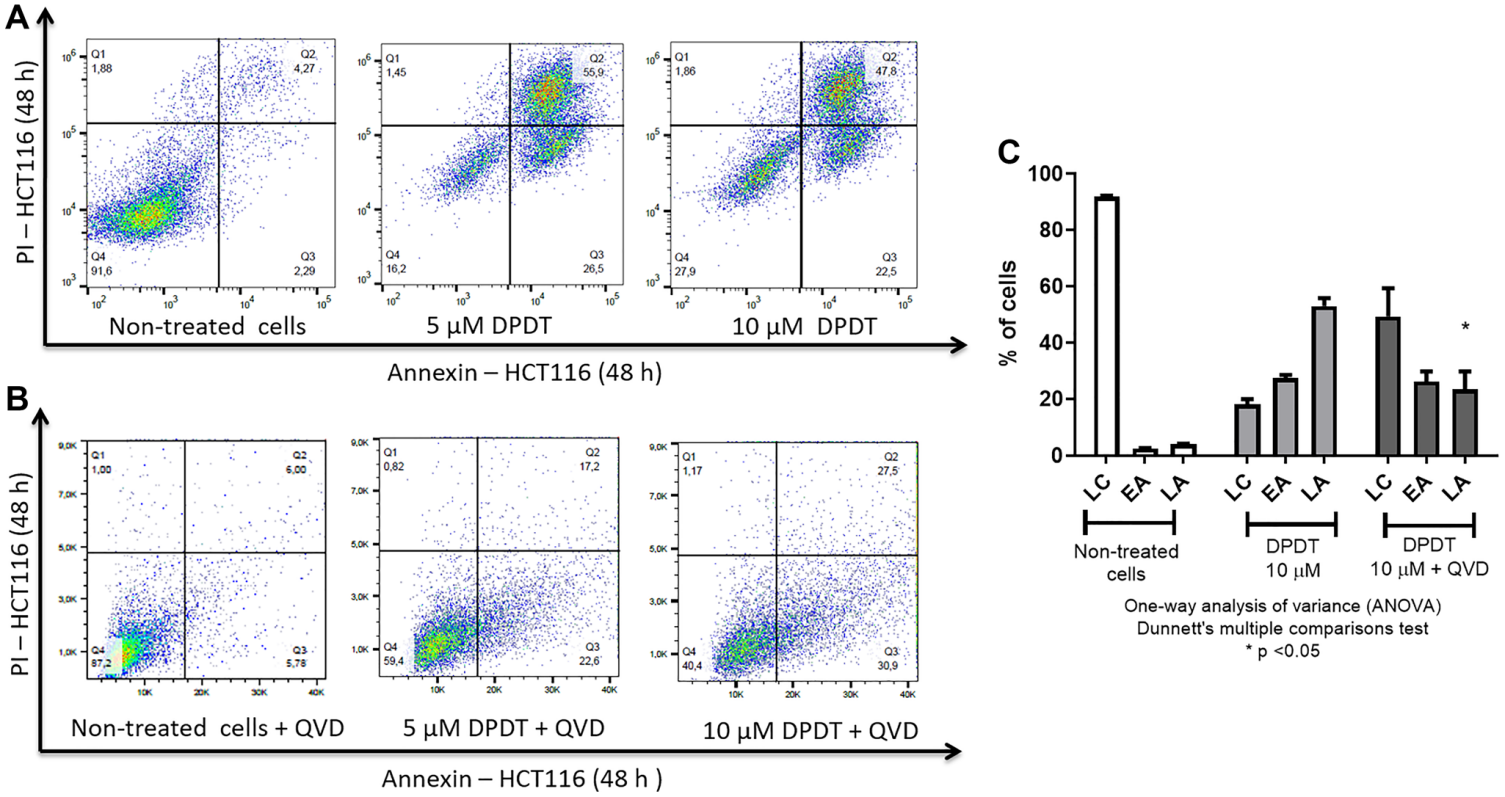
Apoptosis induction by DPDT. (**A**) Representative flow cytometry diagrams obtained for HCT116 cells treated or not with DPDT. HCT116 cells were left untreated or incubated with 5 or 10 μM DPDT for 48 hours. The percentage of cells in Q4 (living cells, annexin V and PI negative cells), Q3 (early apoptotic cells, annexin V positive and PI negative cells) and Q2 (late apoptotic cells, annexin V and PI positive cells) was determined by flow cytometry. (**B**) Representative flow cytometry diagrams obtained for HCT116 cells treated or not with DPDT for 48 hours in presence of the pan-caspase inhibitor QVD-Oph (10 μM). (**C**) Data are expressed as the mean +/− SD (*n* ≥ 3). Abbreviations: LC: Living cells; EA: Early apoptotic cells; LA: Late apoptotic cells. SDs are indicated by error bars when they exceed symbol size. *P* values relative to the QVD-Oph untreated cells were calculated using one-way ANOVA Dunnett's multiple comparison test: ^*^ *p* < 0.05.

Because cell viability and colony formation assays do not allow to strictly distinguishing between a cytotoxic effect and a cytostatic one, we evaluated the capability of DPDT to induce cell death (Figure 2). To evaluate whether the changes in cell viability were associated with a subsequent induction of cell death, HCT116 cells were treated for 48 hours with either 5 or 10 μM DPDT and the percentage of Annexin V/propidium iodide (PI) positive cells determined (Figure 2A, 2C). Our results first confirmed that DPDT can induce both early (annexin V positive, propidium iodide negative cells) and late apoptosis (annexin V positive, propidium iodide positive cells) in HCT116 cells (Figure 2A). Again, this result was confirmed in HeLa cells (Supplementary Figure 2). To further characterize the cell death processes induced by DPDT, HCT116 cells were treated for 48 hours with either 5 or 10 μM of DPDT in presence of QVD-Oph (a pan-caspase inhibitor with potent anti-apoptotic properties) (Figure 2B, 2C). Interestingly, DPDT-induced cell death was markedly inhibited by QVD-Oph in HCT116 cells suggesting that DPDT acts through caspases-mediated apoptosis in these cells.

### Cell cycle modulation in HCT116 and MRC5 cells induced by DPDT

**Figure 3:**
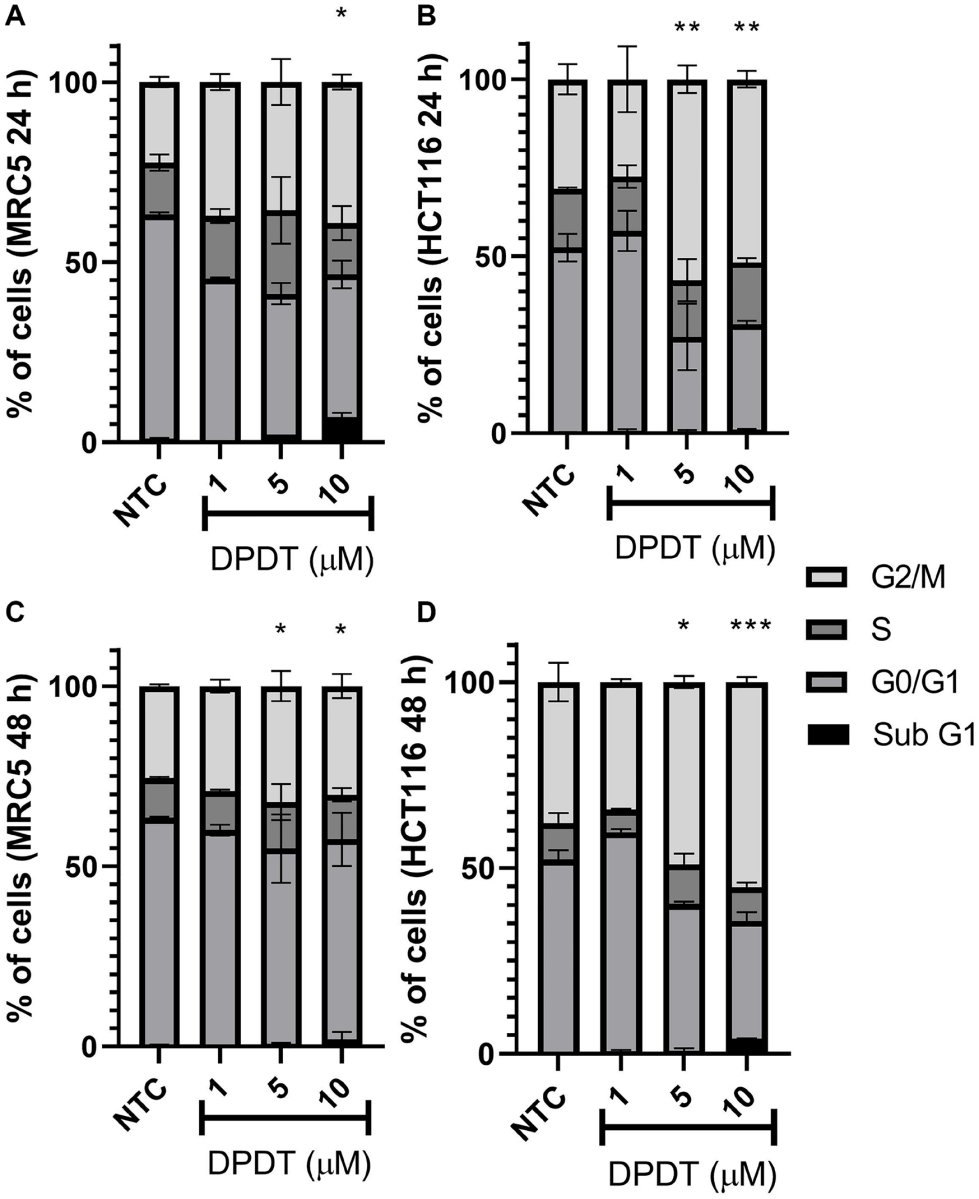
Effect of DPDT on the cell cycle progression of MRC5 and HCT116 cells. Cells were treated for 24 (**A** and **B**, respectively) or 48 hours (**C** and **D**, respectively) with the indicated DPDT concentrations. Histograms show the percentage of cells in G0/G1, S or G2/M. Data are significantly different in relation to the negative control group ^*^ *p* < 0.05; ^**^ *p* < 0.01; ^***^ *p* < 0.001/One Way ANOVA Dunnett's multiple comparison test. ^*^NTC (Non-treated cells).

To determine whether treatment with DPDT affects cell cycle progression in both HCT116 and MRC5 cells, we labeled cells with propidium iodide after 24 and 48 hours of treatment with increasing concentrations of DPDT (Figure 3). Interestingly, while an accumulation of cells in G2/M was observed for both cell lines after treatment with DPDT, a much stronger effect was seen for HCT116 cells. By 24 and 48 hours, more than 50% of HCT116 cells were indeed arrested in G2/M phases after treatment with either 5 or 10 μM DPDT (Figure 3B, 3D) while this percentage only reached 40% and 30% when the MRC5 cells were treated with DPDT for 24 and 48 hours, respectively (Figure 3A, 3C).

### DPDT induces the formation of topoisomerase I-DNA covalent complexes in HCT116 cells

**Figure 4:**
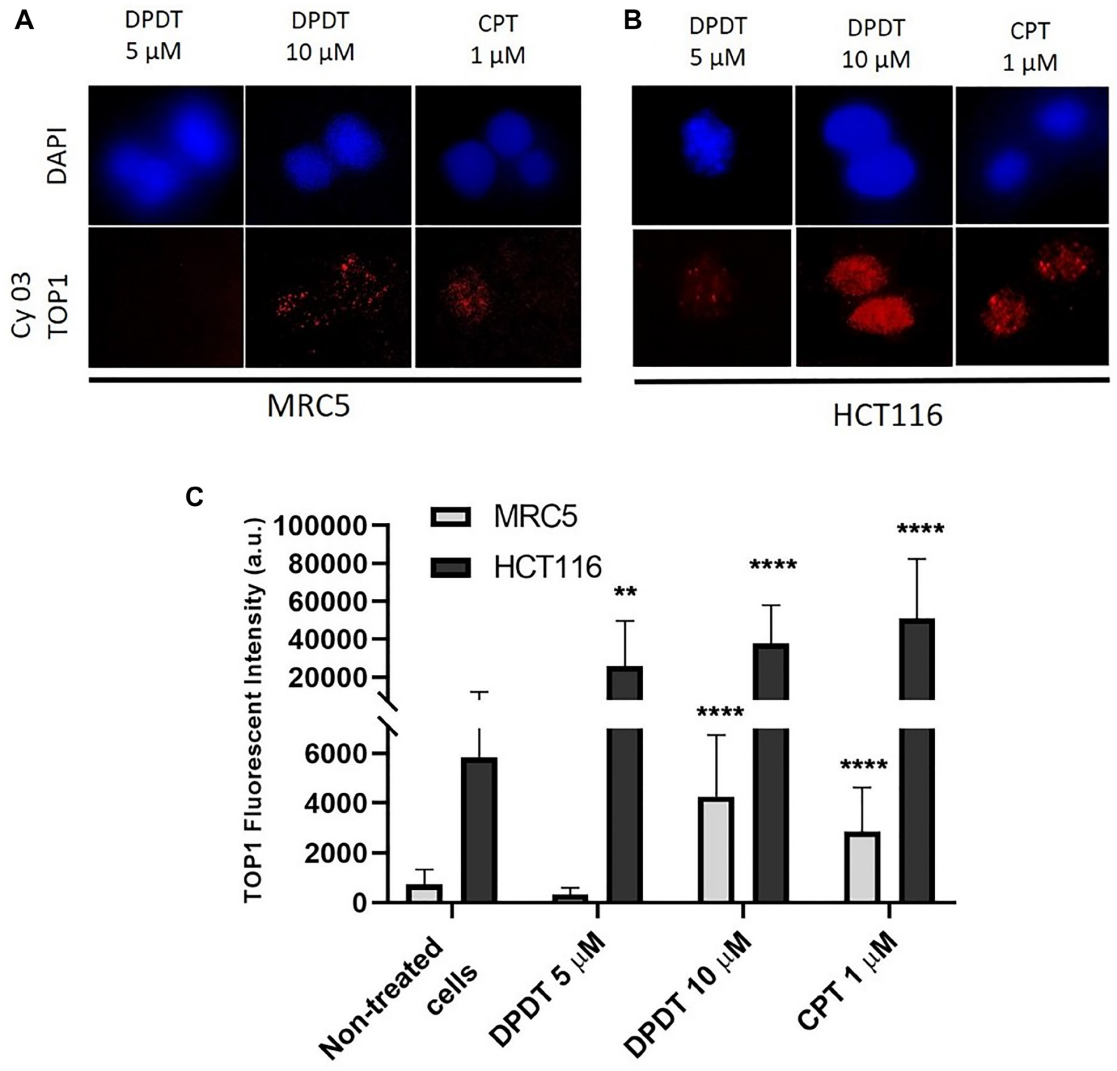
DPDT induces TOP I-DNA complexes in MRC5 and HCT116 cells (TARDIS assay). (**A** and **B**) show imaging of either MRC5 (**A**) or HCT116 (**B**) cells mounted on low melting agarose pre-coated slides after treatment with 0, 5 or 10 μM DPDT for 3 hours. Treatment with 1 μM CPT served as positive control. After drug exposure, cells were labeled with an anti-topoisomerase I antibody and nuclei were counterstained with DAPI. (**C**) Fluorescence intensities were quantified and data are expressed as the mean +/− SD (*n* ≥ 3). *P* values were calculated using One Way ANOVA Dunnett's multiple comparison test (^**^ *p* < 0.01; ^****^ *p* < 0.0001).

Because DPDT has been previously reported to interact with TOP1 in *Saccharomyces cerevisiae* as well as in a cell free system [19, 20], we quantified by using the TARDIS assay the capability of DPDT to poison DNA topoisomerase I in both HCT116 and MRC5 cells (Figure 4). Agarose embedded MRC5 and HCT116 cells were first exposed for 3 hours to 5 or 10 μM of DPDT, as well as to 1 μM of CPT, a well-known topoisomerase I inhibitor. After extraction, cells were then immunolabeled with an anti-topoisomerase I antibody for detecting TOP1-DNA complexes (Figure 4A, 4B). Interestingly, while individual MRC5 cells showed a slight immunostaining after being exposed to 10 μM of DPDT, HCT116 cells showed a marked accumulation of topoisomerase I-DNA complexes at both 5 and 10 μM DPDT. Importantly, the quantification revealed that exposure to 5 μM DPDT increased by more than 3 times the levels of trapped DNA topoisomerase I complexes observed in HCT116 cells while it did not affect the accumulation of topoisomerase I-DNA complexes in MRC5 cells (Figure 4C). Furthermore, when cells were exposed to 10 μM of DPDT, HCT116 cells still demonstrated a more pronounced sensitivity towards DPDT than MRC5 cells. Finally, the effect seen for 10 μM DPDT was very similar to what was observed after 1 μM CPT exposure in both MRC5 and HCT116 cells (Figure 4C). These results suggest that similarly to what was observed in *Saccharomyces cerevisiae*, DPDT is capable to act as a DNA topoisomerase I poison.

### DPDT exposure induces DNA strand breaks in cycling cells

**Figure 5:**
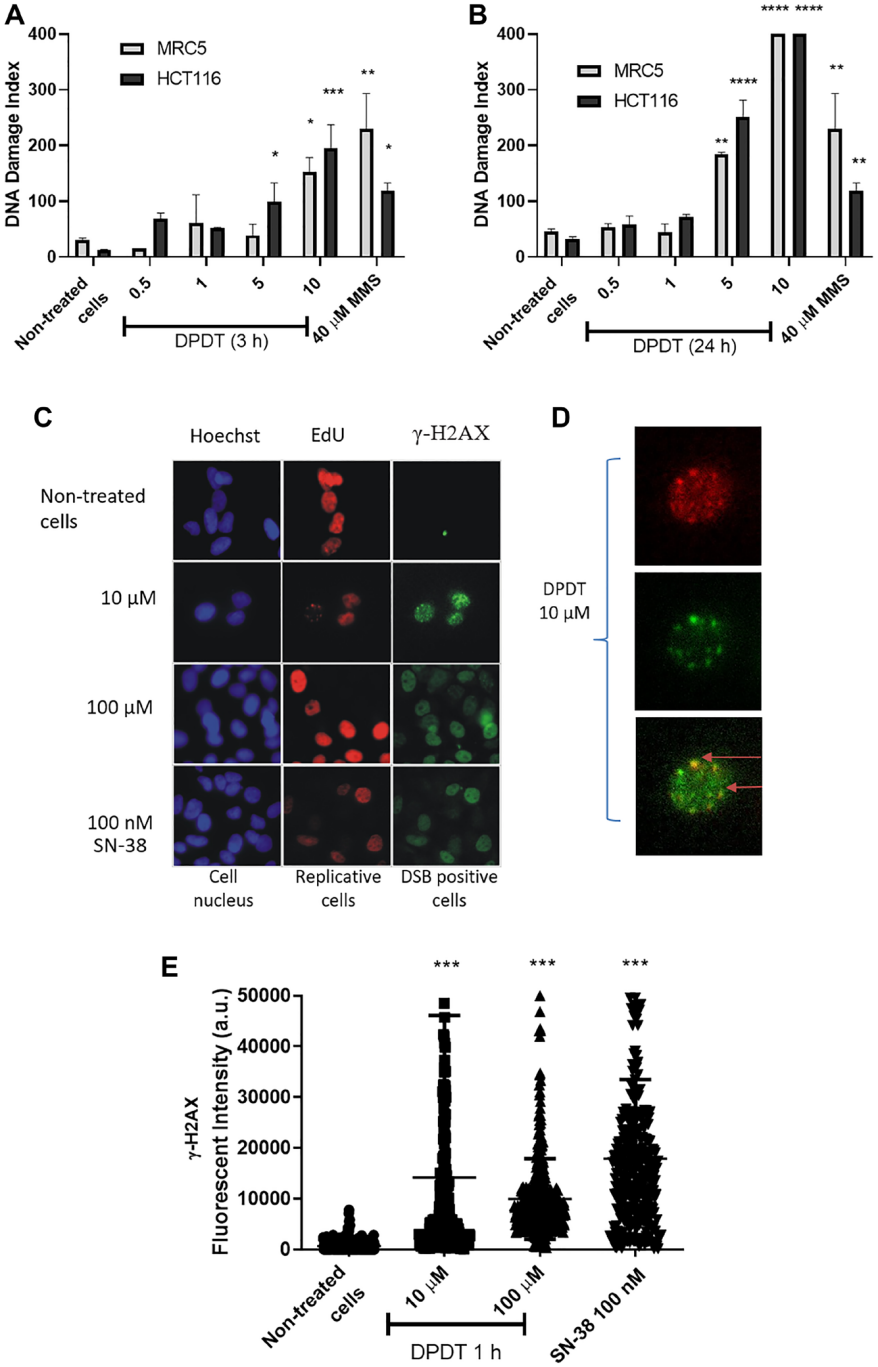
DPDT induces DNA damage in proliferating cells. Proliferating MRC5 and HCT116 cells were treated for 3 hours (**A**) or 24 hours (**B**) with 0, 1, 5, and 10 μM DPDT or 40 μM MMS (3 h) and subjected to alkaline comet assay. (**C**) Proliferating HCT116 cells were incubated with EdU for 30 min followed by 1 hour exposure to 0, 10 or 100 μM DPDT or 100 nM SN-38. Cells were fixed and processed for EdU and γ-H2AX staining, and the DNA was counterstained with Hoechst. (**D**) Increased magnification of the 10 μM DPDT treated cells shown in (C). The merged image illustrates the co-localization of EdU and γ-H2AX (indicated with arrows). (**E**) HCT116 cells were mock-treated or exposed for 1 hour to DPDT (10 or 100 μM) or SN-38 (100 nM). Cells were then fixed and processed for immunolabeling with an antibody directed against γ-H2AX. The fluorescence intensities were quantified and are indicated in arbitrary units (a.u.). At least 100 cells were analyzed for each condition. *P* values relative to the untreated cells were calculated using one-way ANOVA with Dunnett’s multiple comparison test: ^*^ *p* < 0.05, ^**^ *p* < 0.01, ^***^ *p* < 0.001, ^****^ *p* < 0.0001.

Antitumor agents acting as DNA topoisomerase I poison are known to induce DNA breakage at sites where TOPI is covalently bound to DNA. To determine whether DPDT might also exert a genotoxic effect on both HCT116 and MRC5 cells, we first quantified through the alkaline comet assay the ability of DPDT to induce DNA strand breaks. To do so, cells were exposed to 5 μM or 10 μM of DPDT for 3 or 24 hours and DNA breakage formation measured (Figure 5A, 5B). In this setting, DPDT induced a dose-dependent increase of the DNA damage index (DI) for both cell lines. Of note, after 3 or 24 hours of exposure to 5 μM of DPDT, HCT116 cells showed a higher DI than MRC5 cells. However, when cells were exposed to 10 μM of DPDT, the calculated DI were similar for both HCT116 and MRC5 cell lines suggesting that DPDT might exert pejorative effects on normal-like cells when used at high drug concentrations.

Because DNA topoisomerase I inhibitors exert their effect by creating a cleavable nucleoprotein complex able to interfere with the DNA replication process leading to the formation of DNA double-strand breaks (DSBs), we labeled HCT116 cells with EdU, a thymidine analogue, prior to a short-time (1 hour) treatment with DPDT. The sites of DNA double-strand breaks were then visualized through γ-H2AX immunostaining to establish the direct relationship between DSBs induction and the replication status (Figure 5C, 5D). Because of the short exposure time, high DPDT concentrations (10 and 100 μM) were used in this specific setting. Importantly, although no γ-H2AX signal could be observed in the absence of drug, DPDT exposure was accompanied by an important increase in γ-H2AX labeling in HCT116 cells (Figure 5C, 5D). Moreover, the majority of cells that were positive for γ-H2AX were also positive for EdU (Figure 5D and Supplementary Figure 3), whereas almost no detectable γ-H2AX staining was observed for non-replicating cells when 10 μM of DPDT was used. Interestingly, this pattern was very similar to what was observed for SN-38, the active metabolite of CPT (Figure 5D). Further analysis revealed that the DPDT-induced γ-H2AX foci were associated with sites of DNA synthesis within individual cells (Figure 5E). The effect of DPDT was not limited to HCT116 cancer cell line since HeLa cells showed a very similar pattern when they were exposed to the drug (Supplementary Figure 4). Together, these results suggest that the early induction of the γ-H2AX staining by DPDT is mostly limited to actively replicating DNA regions, as it was also reported for other DNA topoisomerase I poisons.

### DPDT induces ROS accumulation in HCT116 and MRC5 cells

**Figure 6:**
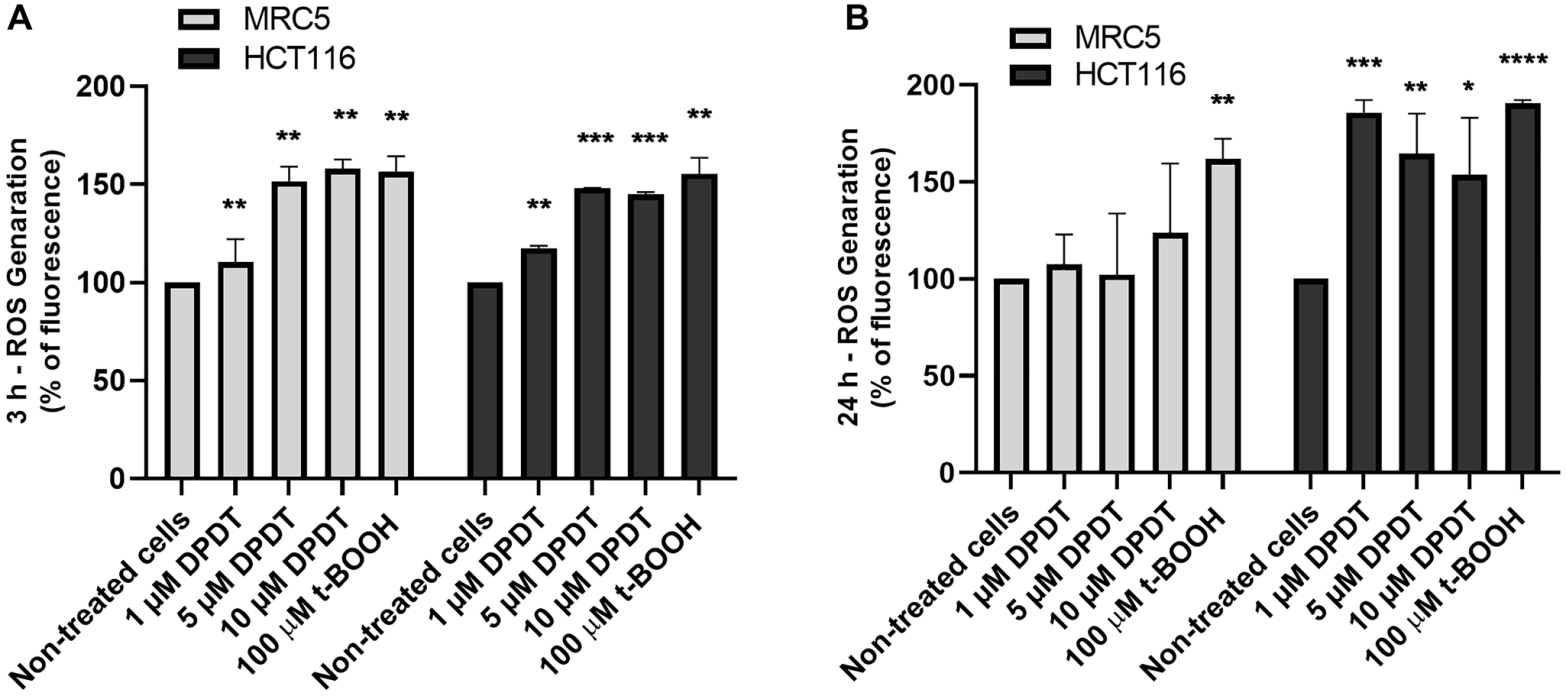
DPDT induces ROS accumulation in HCT116 and MRC5 proliferating cells. Proliferating MRC5 and HCT116 cells were treated for 3 hours (**A**) or 24 hours (**B**) with 0, 1, 5, and 10 μM DPDT or 100 μM t-BOOH and subjected to DCFH-DA fluorescent labeling. The fluorescence intensities were quantified by flow cytometry and are expressed as the percentage of fluorescence increase in relation to the mock-treated cells (mean ± SD), *n* = 3. *P* values relative to the untreated cells were calculated using one-way ANOVA with Dunnett’s multiple comparison test: ^*^ *p* < 0.05, ^**^ *p* < 0.01, ^***^ *p* < 0.001, ^****^ *p* < 0.0001.

While low concentrations of DPDT are believed to exert an antioxidant activity, DPDT can also cause depletion of glutathione (GSH). To determine whether treatment with cytotoxic concentrations of DPDT might increase ROS production in both MRC5 and HCT116 cells, they were incubated with either 5 or 10 μM of DPDT for 3 (Figure 6A) or 24 hours (Figure 6B). Intracellular ROS levels were then measured by flow cytometry by using the cell-permeant DCFH-DA probe. Interestingly, while ROS accumulation increased in both cell lines in a concentration-dependent manner after 3 hours of drug exposure (Figure 6A), ROS levels were back to normal in MCR5 cells after 24 hours. On the contrary, the levels of ROS remained high in HCT116 cells after 24 hours of DPDT exposure. This suggests that MRC5 cells might deal more efficiently than the HCT116 with the ROS produced following exposure to high DPDT concentrations.

## DISCUSSION

The treatment of CRC patients with metastatic disease consists in using cytotoxic chemotherapies, such as oxaliplatin, 5-fluorouracil, capecitabine and irinotecan [1]. The use of those drugs is however limited due to the emergence of resistance which requires novel approaches to overcome the underlying mechanisms [28, 29]. In that sense, the cytotoxic potential of DPDT has been previously evaluated against several tumor cell models, such as CRC, gliomas, melanoma and promyelocytic leukemia cancer cells [22]. This compound was suggested to act as a putative TOP1 inhibitor in *in vitro* studies as well as in yeast models [20]. Several studies also reported genotoxic, mutagenic and pro-oxidant properties of DPDT in different biological models [22, 25]. However, its precise mechanism of action against tumor cells has been to date poorly characterized. Thus, our study aimed at investigating the cytotoxic activity of DPDT in CRC HCT116 cells and to compare it to its effect on the non-tumor MRC5 cell line.

Our results first show that DPDT acts *in cellulo* as a DNA TOP1 poison. These data are in agreement with our previously published report demonstrating that DPDT is capable to inhibit the human TOP1 activity *in vitro* [20]. This hypothesis is also supported by our studies made on *Saccharomyces cerevisiae* demonstrating that TOP1-deficient strains were more tolerant to DPDT treatment than their isogenic wild-type counterpart [20]. A key function of DNA topoisomerase I is relaxing supercoiled DNA ahead of the replication and transcription machinery as well as during DNA repair and chromatin remodeling. Inhibitors of TOP1 block the second transesterification reaction, which prevents DNA religation and stabilizes the cleavable complex drug-DNA-TOP1 [11]. The cytotoxicity of TOP1 poisons has been strongly linked with an increased DNA damage scoring, especially through the formation of DNA double strand breaks (DSBs) in S-phase [11, 30]. The collision of DNA replication forks with the ternary complexes is indeed believed to induce replication-dependent DSBs and to consequently trigger the DNA damage response signaling through histone variant H2AX phosphorylation [11, 31]. Accordingly, we show that DPDT induces the formation of γ-H2AX foci in S-phase in both HCT116 and HeLa cells which strongly, but not exclusively, correlated with the replication sites visualized through EdU staining (Figure 5, Supplementary Figures 3 and 4, respectively). DSBs induction was followed by a strong accumulation of HCT116 cells in G2/M (Figure 3) after 24 or 48 hours of DPDT exposure. Our observations are fully consistent with those from others demonstrating that DPDT induces in several cancer cell lines (e.g., C6 glioma and HL-60 cells) a cell cycle arrest in S or G2/M [22, 23]. These evidences therefore suggest that DPDT can interfere with DNA replication by inhibiting TOP1 and subsequently activate checkpoint kinases leading to a cell cycle arrest in either S or G2/M phases. Remarkably, exposure of MRC5 cells to low concentrations of DPDT (≤ 5 μM) led to a much more modest, if any, effect on cell cycle progression. This observation is in agreement with the lower number of toxic ternary complexes observed in MRC5 cells (Figure 4) which correlates with a lower level of DNA damage observed in those cells when compared to HCT116 cells (Figure 5).

Importantly, the cellular effects of DPDT are linked, not only to TOP1 poisoning, but likely also to its ability to disturb cellular redox homeostasis. In that sense, our results show that DPDT exposure increased ROS generation in both HCT116 and MRC5 (Figure 6). Again, these results are in agreement with previous reports demonstrating that DPDT significantly decreases the GSH/GSSG ratio in HT-29 cells when cells were exposed to high drug concentrations ranging from 62.5 μM to 1000 μM [21]. The reduction of the GSH/GSSG ratio combined with an increased lipid peroxidation were also observed in *S. cerevisiae* and V79 cells after DPDT exposure [24]. However, as for the cell cycle progression, the effect of DPDT on the ROS levels observed in MRC5 cells after 24 hours of drug exposure was markedly decreased when compared to the levels measured in HCT116 cells. This suggests that non-cancer cells might deal more efficiently with ROS and the subsequent oxidative damages induced by DPDT than cancer cells. Interestingly, the use of oxidative agents in the treatment of cancer has been a promising therapeutic strategy. Non-cancer cells are indeed characterized by a low basal level of ROS compared to cancer cells. Likewise, the cancer cells develop an increased antioxidant capacity as a compensatory mechanism to escape the ROS-induced cell death, thus increasing their vulnerability to redox state-modulating agents [32].

The specific nature of cancer cells regarding their ability in dealing with both DNA and oxidative damages might help to develop compounds targeting more selectively tumor cells. In that sense, we show here that DPDT more strongly impacts the cellular viability of the HCT116 and HeLa cells when compared to MRC5 cells (Figure 1 and Supplementary Figure 1). In particular, at low drug concentration, the effect of DPDT was more intense on HCT116 cells compared to the non-tumorigenic MRC5 cell line suggesting a possible therapeutic window for DPDT (Figure 1). These results confirmed the preferential effect of DPDT on cancer cells that was already demonstrated by others when compared to normal human peripheral blood mononuclear cells [22]. Importantly, our results show that this effect is not only due to a cell cycle arrest (Figure 3) but also to apoptosis induction (Figure 1) through a caspase-dependent pathway (Figure 2). This confirms our previous observation, as well as those of Sailer and collaborators, demonstrating that DPDT exposure induced apoptosis in V79 and HL-60 cells, respectively [20, 23].

Altogether, our results show that DPDT preferentially targets HCT116 colon cancer cells likely through DNA topoisomerase I poisoning. This makes DPDT an interesting molecule for further development as an anti-proliferative compound in the context of cancer.

## MATERIALS AND METHODS

### Chemicals

DPDT (CAS: 32294-60-3) was purchased from Sigma Aldrich. Dulbecco’s modified Eagle Medium (DMEM), fetal bovine serum (FBS), trypsin-EDTA, L-glutamine, and antibiotics were purchased from Gibco BRL (Grand Island, NY, USA); methyl methanesulfonate (MMS) and camptothecin (CPT), SN-38, dimethyl sulfoxide (DMSO) and 3-(4,5-dimethylthiazole-2-yl)-2,5-biphenyl tetrazolium bromide (MTT) were from Sigma (St. Louis, MO, USA). Low-melting point agarose and normal agarose were obtained from Invitrogen (Carlsbad, CA, USA). Propidium Iodate (PI) was purchased from Thermo Fisher. Giemsa stain was from Merck (Darmstadt, Germany) and 2′,7′-dichlorfluorescein-diacetate (DCFH-DA) from Invitrogen (PoortGebouw, The Netherlands). All other reagents were of analytical grade. The tissue culture flasks (bottles and dishes) were supplied by TPP (Trasadingen, Switzerland). Quinoline-Val-Asp-Difluorophenoxymethylketone (QVD-OPh) were purchased from Abcam^®^ (ab141421).

### Antibodies

The γ-H2AX (#05-636) antibody was purchased from Millipore. Click-iT™ EdU (5-ethynyl-2’-deoxyuridine) Cell Proliferation Kit for Imaging, Alexa Fluor™ 555 dye was purchased from ThermoFisher (C10338). Human Anti-Topoisomerase I antibody was purchased from Abcam (ab3825).

### Cell culture and treatments

Human fetal lung fibroblast (MRC5) and colon carcinoma (HCT116) cells were purchased from American Type Culture Collection (ATCC). The cell culture was performed as described by Masters and Stacey with minor modifications [33]. Cells were grown in Eagle’s modified Dulbecco culture medium (DMEM), pH 7.4, supplemented with 10% inactivated fetal bovine serum, glutamine, 0.2 mg/ml penicillin, 100 μg/mL streptomycin and preserved in 25 cm^3^ culture flasks at 37°C and humidified atmosphere containing 5% CO_2_. For harvesting and culture establishment, cells were washed with phosphate buffer saline (PBS) pH 7.4 and incubated with 0.15% trypsin-0.08% EDTA. Cells were seeded in complete medium and grown to 50–60% or 80–90% confluence depending the exposure time, prior to the treatment with the test substance. All cell lines were regularly tested for *Mycoplasma* contamination using *Mycoplasma* Detection Kit Myco Alert^®^ Antibodies (Lonza).

The DPDT was prepared in DMSO to reach all concentrations and finally an appropriated amount was added to DMEM to achieve the different designed concentrations, all with the same final percentage of DMSO. Cells were treated with DPDT for 3, 24, 48 or 72 hours at concentrations from 0.1 to 20 μM DPDT in DMEM culture, then washed with PBS at pH 7.4 and submitted to tests. The final DMSO concentration in the medium never exceeded 0.5%, and the control group was exposed to an equivalent concentration of solvent.

### MTT assay

MTT assay was performed as described by Mosmann (1983) with minor modifications [34]. Briefly, the cell culture was established in 24 well plates, in which were seeded 3 × 10^4^, 5 × 10^4^ or 6 × 10^4^ cells per well according to the exposure time. After the exposures (3, 24, 48 or 72 h), the cells were washed once with PBS before adding 0.5 mL serum-free medium containing tetrazolium salt MTT dye 1 mg/mL to each sample. After incubation for 3 hours, the supernatant was removed, and the obtained purple formazan product was dissolved in 0.5 mL of DMSO stirred for 10 min, and the absorbance was measured using a microplate reader (Perkin Almer – Inspire^®^, USA) at 570 nm. Results were expressed as mean percentage of absorbance in treated cells as compared to negative control (considered 100%). The IC_50_ (concentration that inhibits cell growth by 50%) ratio of tumoral (HCT116) and non-tumor (MRC5) cell was also calculated.

### Colony-formation assay (clonal survival)

The clonal survival was performed as described by Franken (2006) with minor modifications [35]. Cells were plated onto 6-well plates at a concentration of 200 cells per well. Cells were maintained in DMEM media culture with FBS for 24 hours at 37°C and 5% CO_2_ and then were exposed to different concentrations of DPDT for 72 hours. After treatment (72 hours), the media culture containing the test substance was removed and the cells were washed with PBS and re-incubated with drug free media for 7 to 10 days. After, the media were aspirated, the cells were fixed with ethanol and stain with 5% Giemsa solution. The colonies containing more than 50 cells were counted and the survival was calculated as a percentage relative to the number of colonies of the negative control (considered 100%).

### ROS levels determination by flow cytometry analysis

The levels of intracellular ROS were determined by conversion of 2′,7′-dichlorodihydro-fluorescein diacetate (DCFH-DA) to the highly fluorescent 2′,7′-dichlorofluorescein in the presence of oxidant. Briefly, the cells were grown overnight and treated with DPDT for 3 or 24 hours. After that, the cells were washed and incubated with 10 μM DCFH-DA for 30 min. After incubation, the cells were washed, harvested and evaluated by flow cytometry (Guava^®^ EasyCyte cytometer - EMD Millipore, Germany) with measurement of 10,000 cells per experimental condition and FlowJo software (BD Bioscience). The ROS production was expressed as a relative change of fluorescence accumulation in the treated cells in relation to the controls.

### Alkaline comet assay

To evaluate the genotoxicity, the comet assay or single cell gel electrophoresis was used, which measures DNA damage as described by Singh et al. (1988) with minor modifications, where culture was established with 6 × 10^4^ cell per well in 24 well plates [36]. One hundred nucleoids of each treatment are assessed visually and categorized into five classes of damage: (i) damage 0: with no tail; (ii) damage 1: the tail is less than the diameter of the head; (iii) damage 2: the length of the tail is 1, 2 times the diameter of the head; (iv) damage 3: the tail is greater than twice the diameter of the head; (v) damage 4: comets without a clear head. The results are presented as DNA damage index (DI), ranging from 0 (100 undamaged cells) to 400 (100 cells with damage 4). Visual analysis of the comet assay is an internationally accepted method and has a significant correlation with computerized analysis [37, 38]. The vehicle was used as a negative control, MMS treatment at 4 × 10^−5^ M for 3 hours was used as a positive control.

### γ-H2AX analysis

Cells were incubated with 10 and 100 μM for one hour and SN-38 was used as positive control. Before exposure, cells are incubated with EdU (Click- iT^TM^ EdU Alexa Fluor^®^ 555 Imaging Kit, #C10338, Invitrogen) for 30 min. After, the cells were labeled with γ-H2AX (antibody) for detection of DSB in replicating cells. Collection of images were made in BX61 microscope and cell F imaging software (Olympus). Fluorescence quantification analysis was made in ImageJ software. At least 100 cells were analyzed and the representative means value are of at least two experiments.

### Cell cycle progression analysis

Cell cycle distribution on DPDT exposure was assessed using flow cytometry. Briefly, 6 × 10^4^ cells were cultured in 24-well plate and exposed to 1, 5 or 10 μM DPTD for 24 or 48 hours. After exposure, cells were stained with PSSI solution (Triton X-100 0.1%, RNAse 0.5 mg/mL and PI 6 lM per sample, in PBS). Cell samples were analyzed (10,000 events) with a Guava^®^ EasyCyte cytometer (EMD Millipore, Germany) and Flow Jo software.

### Apoptosis analysis

Cell culture was established at 5 × 10^4^ and after 72 hours exposure, the cells were incubated with propidium iodide for 30 min and submitted to 10,000 events analyses in the Guava^®^ EasyCyte cytometer (EMD Millipore, Germany). The same incubation was realized with QVD-OPh (a caspase-3, 1, 8 and 9 inhibitor), for 30 min and following DPDT exposure.

### Trapped in agarose DNA immunostaining (TARDIS) assay

The TARDIS assay was performed as described by Wilmore et al. (1998) with minor modifications [39]. Briefly, HCT116 and MRC5 were seeded in 5 × 10^4^ cells per well, in a 24-well plate exposed to 5 and 10 μM for 3 hours followed by mounting in pre-coated slide with agarose low-melting. Right after, cells were incubated with anti-TOP1 antibody (1:200), cyanine 3 (Cy3) (conjugated secondary antibody) and DAPI. Fluorescence images were captured with a fluorescence microscope OLYMPUS BX51 (Olympus Corporation, Tokyo, Japan) and the areas occupied by the blue and red fluorescence were quantified with ImageJ software (NIH, Maryland, USA). To measure the capacity for removal of TOP1 DNA complexes cells were exposed to DPDT or CPT, as positive control. Thus, approximately 100 cells were analyzed for each experimental condition.

### Statistical analysis

The data were obtained from at least three independent experiments in duplicate samples for each treatment. Results are expressed as mean ± standard deviation (SD). Data were analyzed by one-way analysis of variance (ANOVA), followed by test of Tukey or Dunnett’s multiple comparison test with *p* < 0.05 considered as statistically significant.

## SUPPLEMENTARY MATERIALS



## Abbreviations

5-FU: 5-fluorouracil
CPT: camptothecin
CRC: Colorectal cancer
DMEM: Dulbecco’s Modified Eagle Medium
DMSO: Dimethyl sulfoxide
DCFH-DA: 2′,7′-dichlorofluorescein diacetate
DPDT: Diphenyl ditelluride
EdU: 5-ethynyl-2′-deoxyuridine
FOLFIRI: Leucovorin, 5-fluorouracil and irinotecan
FOLFOXIR: Leucovorin, 5-fluorouracil, oxaliplatin and irinotecan
GSH: Glutathione
mCRC: metastatic colorectal cancer
MMS: methyl methanesulfonate
OT: Organotellurium
PI: Propidium iodide
QVD-OPh: Quinoline-Val-Asp-Difluorophenoxymethylketone
ROS: Reactive oxygen species
TOP1: Topoisomerase I
TARDIS: Trapped in agarose DNA immunostaining
