# Supplementary material for: Diphenyl ditelluride anticancer activity and DNA topoisomerase I poisoning in human colon cancer HCT116 cells
Source: Oncotarget. 2023 Jun 21;14:637–49. doi: 10.18632/oncotarget.28465 (PMC10284427; doi:10.18632/oncotarget.28465)
Supplement: Supplementary file 1 [file oncotarget-14-28465-s001.pdf]

Diphenyl ditelluride anticancer activity and DNA topoisomerase I poisoning in human colon cancer HCT116 cells

SUPPLEMENTARY MATERIALS

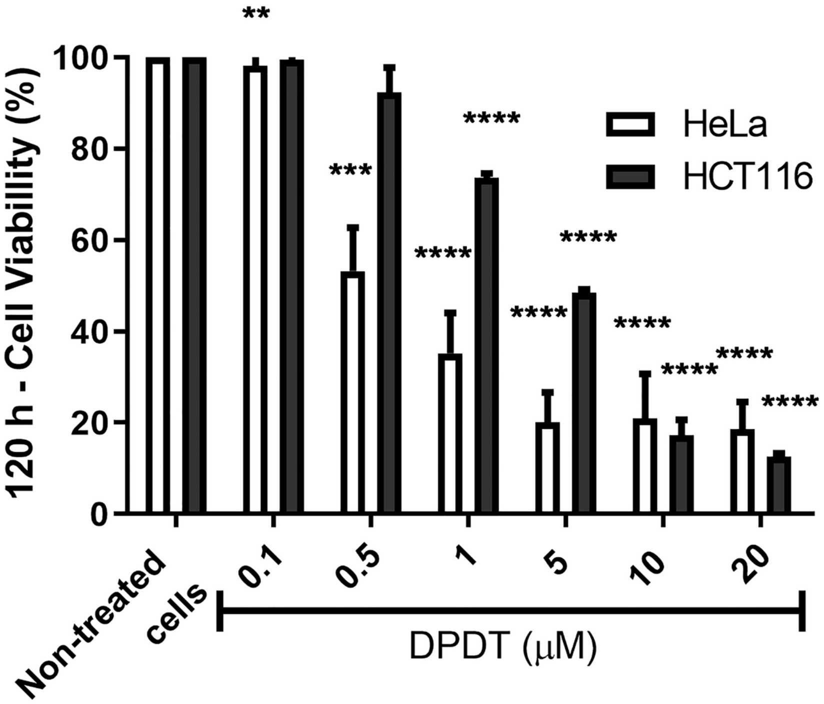

Supplementary Figure 1: Dose-dependent cell viability of DPDT on HeLa and HCT116 cells as determined by the MTT assay. Cells were treated with 0 (Non-treated) or 0.1 to 20 μM DPDT for 72 h. Data are expressed as the percentage of viable cells when compared to the untreated cells (Mean ± S.D.), $n = 3$. P values relative to the control cells were calculated using one-way ANOVA Dunnett's multiple comparison test: $^{*}p < 0.05$, $^{**}p < 0.01$, $^{***}p < 0.001$, $^{****}p < 0.0001$.

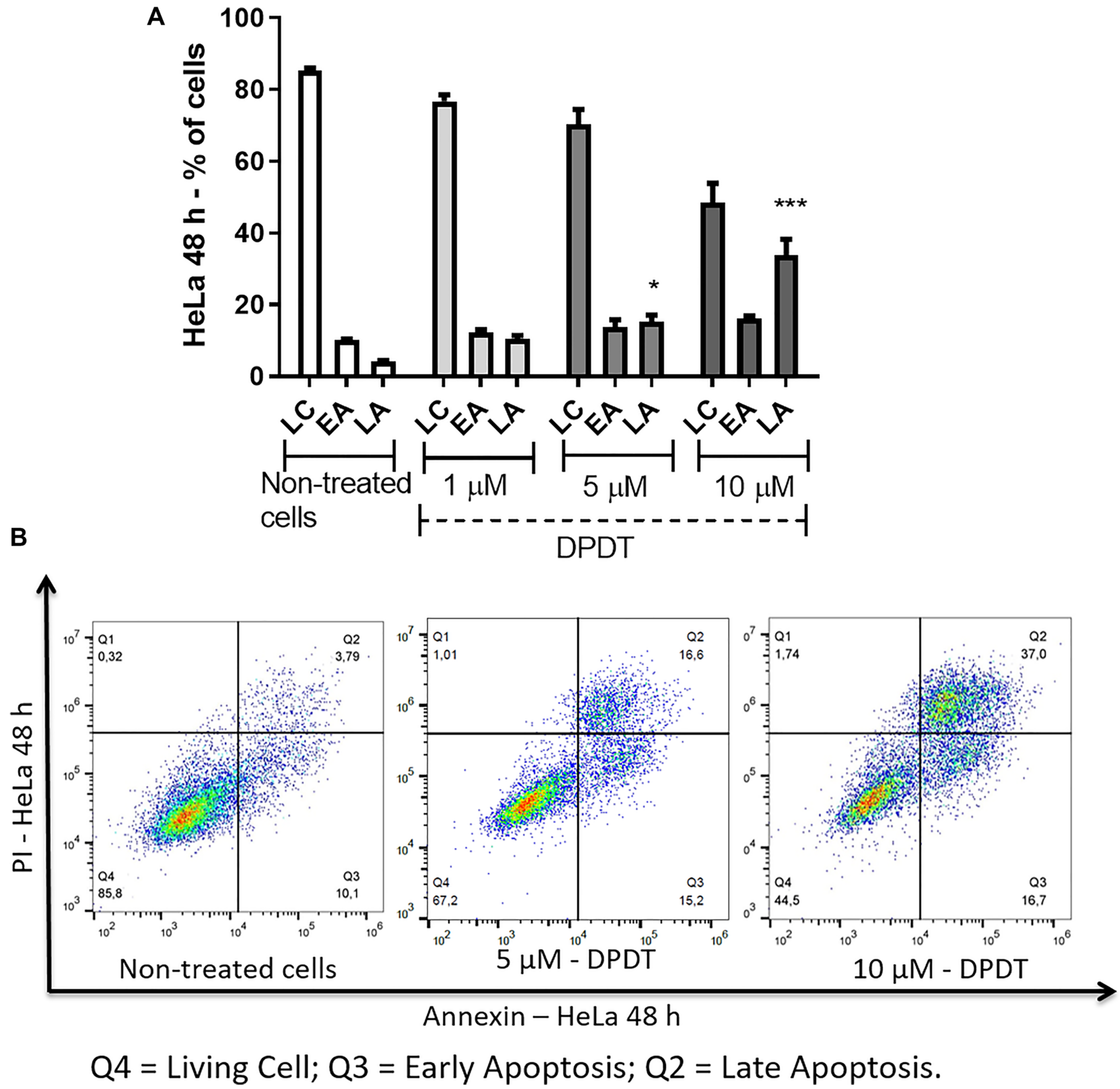

Supplementary Figure 2: (**A**) Bar graphs representing the percentage of HeLa living cells (LC), HeLa cells in early apoptosis (EA) or in late apoptosis (LA) after treatment with 0, 1, 5, 10 μM of DPDT for 48 h. Data are expressed as the mean +/−; SD. *P* values relative to the untreated cells were calculated using one-way ANOVA Dunnett's multiple comparison test: $^{*}p < 0.05$, $^{**}p < 0.01$, $^{***}p < 0.001$, $^{****}p < 0.0001$. (**B**) Representative histogram of HeLa cells labeled with annexin/PI after treatment with 0, 5 or 10 μM of DPDT for 48 h. Q4 represents non-apoptotic HeLa cells, Q3 shows HeLa cells in early apoptosis while Q2 shows cells in late apoptosis.

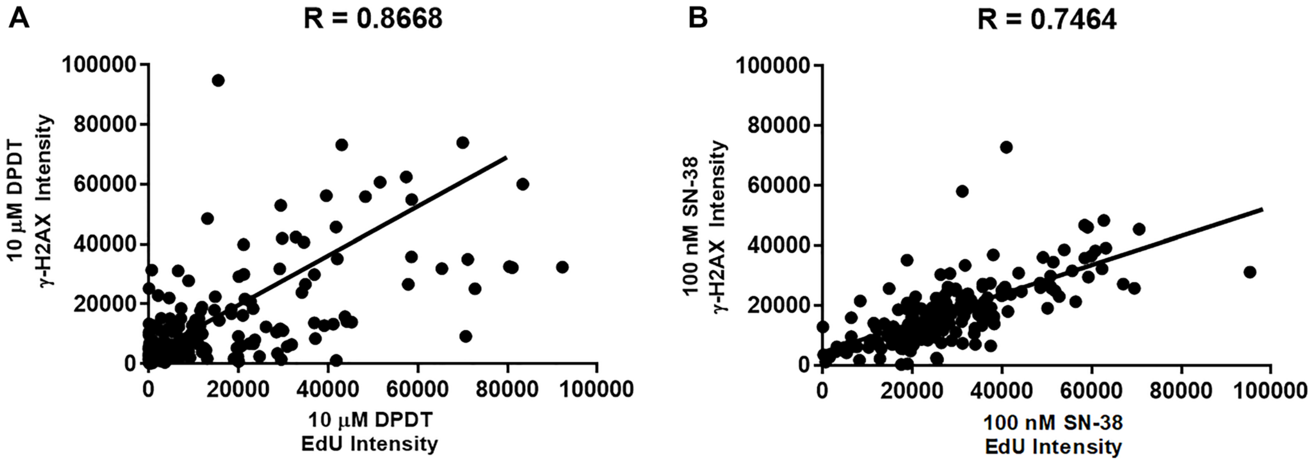

Supplementary Figure 3: Proliferating HCT116 cells were incubated with EdU for 30 min followed by 1 hour exposure to 10 µM DPDT (**A**) or 100 nM SN-38 (**B**). Cells were then fixed and processed for staining of EdU and γ-H2AX. The fluorescence intensities were quantified and are indicated in arbitrary units (a.u.). R denotes the correlation coefficient between the intensities of EdU and γ-H2AX staining.

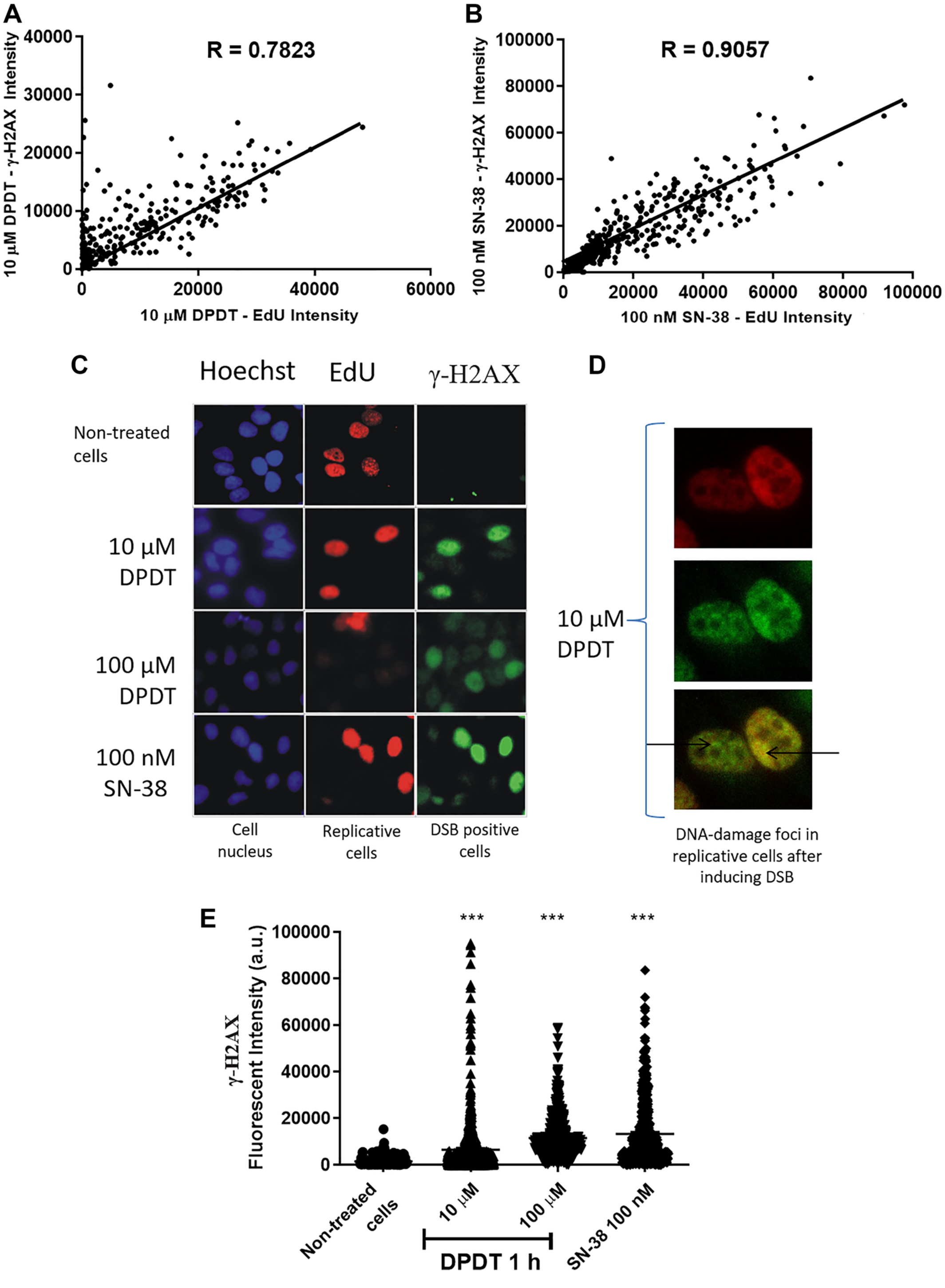

Supplementary Figure 4: (**A** and **B**) Proliferating HeLa cells were incubated with EdU for 30 min followed by 1 hour exposure to 10 µM DPDT (A) or 100 nM SN-38 (B). Cells were then fixed and processed for staining of EdU and γ-H2AX. The fluorescence intensities were quantified and are indicated in arbitrary units (a.u.). R denotes the correlation coefficient between the intensities of EdU and γ-H2AX staining. (**C**) Representative images of proliferating HeLa cells incubated with EdU for 30 min followed by 1 hour exposure to 0, 10 or 100 µM DPDT or 100 nM SN-38. Cells were fixed and processed for EdU and γ-H2AX staining, and the DNA was counterstained with Hoechst. (**D**) Increased magnification of the 10 µM DPDT treated cells shown in (C). The merged image illustrates the co-localization of EdU and γ -H2AX (indicated with arrows). (**E**) HeLa cells were mock-treated or exposed for 1 hour to DPDT (10 or 100 µM) or SN-38 (100 nM). Cells were fixed and processed for immunolabeling with an antibody directed against γ-H2AX. The fluorescence intensities were quantified and are indicated in arbitrary units (a.u.). At least 100 cells were analyzed for each condition. *P* values relative to the untreated cells were calculated using one-way ANOVA Dunnett's multiple comparison test: $^{*}p < 0.05$, $^{**}p < 0.01$, $^{***}p < 0.001$, $^{****}p < 0.0001$.
